# The Recurrence of Symptoms After Anterior Cervical Discectomy and Fusion

**DOI:** 10.7759/cureus.39300

**Published:** 2023-05-21

**Authors:** Basil A Alzahrani, Faisal S Alsharm, Hassan K Salamatullah, Hani H Sulimany, Mohammed A Kashab, Muhammad A Khan

**Affiliations:** 1 Orthopedics, King Saud Bin Abdulaziz University for Health Sciences College of Medicine, Jeddah, SAU; 2 Orthopedics, King Saud Bin Abdulaziz University for Health Sciences College of Medicine, Jeddah, SAU, Jeddah, SAU; 3 Orthopedic Surgery: Orthopedic Spine Surgery and Arthroscopy, King Abdulaziz Medical City / Ministry of National Guard - Health Affairs, Jeddah, SAU; 4 Medical Education, King Saud Bin Abdulaziz University, Jeddah, SAU

**Keywords:** symptoms, recurrence, fusion, discectomy, cervical, acdf

## Abstract

Objectives

This study aimed to evaluate the recurrence symptoms rate after anterior cervical discectomy and fusion (ACDF) for one year and seek the common cervical vertebral disk affected in a tertiary center in Saudi Arabia over the past five years.

Methods

This is a single-center, cross-sectional study conducted on patients followed in our center from January 2016 to December 2022. All patients who were older than 18 and underwent ACDF were included.

Results

Out of 77 patients, 43 (55.8%) have experienced a recurrence of symptoms after the ACDF operation. The highest rate of recurrent symptoms was neck pain 22 (28.6%), left upper limb numbness 20 (26%), and right upper limb numbness 16 (20.8%). It was found that shoulder pain recurred after one level of ACDF in six patients out of 10 (60%), and only one (10%) patient experienced shoulder pain after two-level ACDF.

Conclusion

ACDF has a high rate of recurrence of symptoms, and the most common type of ACDF was two levels. Most symptoms were neck pain and upper limb radicular pain. However, there is a lack of studies. We recommend conducting more studies on the secondary management of recurrent symptoms post-ACDF.

## Introduction

The spinal column comprises cervical, thoracic, lumbar, and sacral vertebrae. Intervertebral discs (IVDs), consisting of an inner gelatinous material called the nucleus pulposus surrounded by outer annulus fibrosis, occur between these vertebrae. The IVD maintains spine mobility and acts as a shock absorber [1,2]. Cervical disc injuries are characterized by the disruption of the IVD structure. The two most common cervical disc pathologies are degenerative disc diseases and herniations. Cervical degenerative disc disease results from reduced water composition in the IVD, whereas cervical disc herniation occurs due to acute traumatic injury to the neck or aging [1,2]. Neck pain is the most frequent presentation of cervical disc injuries. Magnetic resonance imaging is the preferred imaging modality for these conditions. Treatment options include conservative and surgical management. Conservative management includes nonsteroidal anti-inflammatory drugs, steroids, medial bundle branch blocks, collar immobilization, and traction. Surgical procedures are indicated when medical management fails. Surgical options include anterior cervical discectomy and fusion (ACDF), posterior lamina foraminotomy, and cervical disc arthroplasty [1,2].

ACDF is the gold-standard surgical approach for treating cervical disc conditions [3]. Its main indications include cervical disc herniation, degenerative disc disease, and cervical spondylosis [3,4]. Briefly, the procedure is performed by making an incision in the neck anteriorly directly at the level of the injured cervical spine for easy access. The affected disc is removed, and the adjacent vertebrae are fused [4].

Due to its relatively low complication rate, ACDF is considered safe [5,6]. However, several serious complications can persist for long [5]. Dysphagia, hematoma, progressive myelopathy, recurrent laryngeal nerve palsy, cerebrospinal fluid leaks, wound infection, radiculopathy, Horner’s syndrome, respiratory insufficiency, esophageal perforation, and instrument failure are the most frequently reported ACDF-associated complications [7]. Multiple conditions, such as older age and longer operative time, are risk factors for an increased incidence of postoperative complications [8]. Some complications may lead to reoperation after ACDF. As previously reported, the early reoperation rate, mainly due to a postoperative hematoma, was 2.1%, whereas the late reoperation rate, mainly due to adjacent segment disease, was 3.6% [9].

This study aimed to evaluate the symptom recurrence rate one year after ACDF and identify the common cervical vertebral disk affected in a tertiary care center in Saudi Arabia.

## Materials and methods

This single-center, cross-sectional study included patients aged >18 years who underwent ACDF and were followed up at a tertiary center between January 2016 and December 2022. The study was approved by the center’s Institutional Review Board (IRB), approval number NRJ22J/215/08 by King Abdullah International Medical Research Center. Patients who underwent posterior cervical decompression, disc replacement, or previous surgery in the cervical spine were excluded. All eligible patients (n=77) who met the inclusion criteria were included in the study.

Age, sex, operation type, cervical level, and patient symptoms before and after surgery were some of the factors obtained from the patient’s computerized medical records and entered into the data collection sheet. Data were analyzed using the IBM Statistical Package for Social Sciences (IBM Corp., Armonk, NY) version 20.0. Categorical variables were presented as frequency and percentages while continuous variables as mean ± standard deviation (or median and interquartile range as appropriate). When comparing categorical variables, the chi-square test, Fisher’s exact test, or McNemar’s test was used as needed. Statistical significance was set at P<0.05.

## Results

Seventy-seven patients were eligible based on the inclusion and exclusion criteria. Approximately half of the patients were males (39; 50.6%). The mean patient age was 53.1 years. Diabetes mellitus and hypertension were the most prevalent comorbidities among the included patients (31 (40.3%) and 32 (41.6%) patients, respectively). Furthermore, the median time to symptom recurrence after ACDF surgery was six months. Most ACDF surgeries were performed at the C5 and C6 cervical spine levels in 62 (80.5%) and 57 (74%) patients, respectively. However, no ACDF procedures were performed at the level of the first cervical spine, and only one (1.3%) was done at the C2 level.

Most patients (20; 26%) underwent ACDF. Spinal spondylosis 15 (19.5%) and cervical disc herniation 13 (16.9%) were common indications for an ACDF operation in the included patients. The type of ACDF operation was divided into one-, two-, and more than two-level ACDF. Thirty-five (45.5%) patients underwent two-level ACDF while 21 (27.3%) patients underwent one-level or more than two-level ACDF. Demographic data are presented in Table 1.

**Table 1:** Basic characteristics of the patients ACDF: anterior cervical discectomy and fusion

Variable	N= 77
Age (mean ± SD)	53.1±11.33
Gender (n = 77)	
Female	38 (49.4%)
Male	39 (50.6%)
BMI (mean ± SD)	31.3±5.71
Comorbidities (n = 77)	
Diabetes mellitus	31 (40.3%)
Hypertension	32 (41.6%)
Ischemic heart disease	8 (10.4%)
Cancer	4 (5.2%)
Smoker	15 (19.5%)
Time of symptoms recurrence by months (median and IQR)	6 (3-11.5)
Level of ACDF (n = 77)	
C1	0 (0%)
C2	1 (1.3%)
C3	19 (24.7%)
C4	39 (50.6%)
C5	62 (80.5%)
C6	57 (74%)
C7	20 (26%)
Reason for ACDF	
Degenerative disc disease	20 (26%)
Spinal spondylosis	15 (19.5%)
Cervical disc herniation	13 (16.9%)
Spinal stenosis	10 (13%)
Cervical disc prolapse	8 (10.4%)
Cervical fracture	4 (5.2%)
Cervical myeloma	3 (3.9%)
Spinal stenosis + degenerative disc disease	1 (1.3%)
Spinal stenosis + spinal spondylosis	1 (1.3%)
Cervical spondylopathies	1 (1.3%)
Spinal synostosis	1 (1.3%)
Type of operation	
One-level ACDF	21 (27.3%)
Two-level ACDF	35 (45.5%)
> Two-level ACDF	21 (27.3%)

​​Of the 77 patients, 43 (55.8%) experienced complaint recurrence after ACDF. There was no significant association between recurrence, demographic data, and ACDF operation type. However, those who experienced symptom recurrence tended to be older (mean age, 54.2 years) than patients with no symptomatic recurrence after ACDF (mean age, 51.6 years). Moreover, patients with recurrent symptoms had a higher body mass index (mean, 32.1 kg/m2) than those with no recurrent symptoms (mean, 30.3 kg/m2). One- and more than two-level ACDF showed a higher recurrence rate than the two-level ACDF (Table 2).

**Table 2:** Recurrence of symptoms after ACDF by the basic characteristics of patients *chi-squared test; **Fisher’s exact test ACDF: anterior cervical discectomy and fusion

Recurrence				
		Yes		No		P-value
		n=43	%	n=34	%	
Age (Mean ± SD)						0.312
		54.2±10.70		51.6±12.07		
BMI (Mean ± SD)						0.161
		32.1±5.66		30.3±5.69		
Gender						0.414
	Male	20	51.3	19	48.7	
	Female	23	60.5	15	39.5	
Smoker						0.827*
	No	35	56.5	27	43.5	
	Yes	8	53.3	7	46.7	
HTN						0.384*
	No	27	60.0	18	40.0	
	Yes	16	50.0	16	50.0	
IHD						>0.99**
	No	38	55.1	31	44.9	
	Yes	5	62.5	3	37.5	
DM						0.884*
	No	26	56.5	20	43.5	
	Yes	17	54.8	14	45.2	
Cancer						0.316**
	No	42	57.5	31	42.5	
	Yes	1	25.0	3	75.0	
Type of Operation						0.502
	One-level ACDF	13	61.9	8	38.1	
	Two-level ACDF	17	48.6	18	51.4	
	> Two-level ACDF	13	61.9	8	38.1	

All symptoms had significantly lower recurrence rates after ACDF (Table 3). The symptoms with the highest recurrence rates after ACDF were neck pain, left upper limb numbness, and right upper limb numbness in 22 (28.6%), 20 (26%), and 16 (20.8%) patients, respectively. In contrast, the latter symptoms markedly decreased after ACDF compared to before ACDF (P<0.001).

**Table 3:** Recurrence of symptoms before and after ACDF ACDF: anterior cervical discectomy and fusion

Before ACDF		n=77	%	After ACDF		n=77	%	p-value
Neck pain				Neck pain				<0.001
	No	7	9.1		No	55	71.4	
	Yes	70	90.9		Yes	22	28.6	
R upper limb numbness				R upper limb numbness				<0.001
	No	32	41.6		No	61	79.2	
	Yes	45	58.4		Yes	16	20.8	
L upper limb numbness				L upper limb numbness				<0.001
	No	34	44.2		No	57	74.0	
	Yes	43	55.8		Yes	20	26.0	
R upper limb pain				R upper limb pain				<0.001
	No	41	53.2		No	65	84.4	
	Yes	36	46.8		Yes	12	15.6	
L upper limb pain				L upper limb pain				0.002
	No	47	61.0		No	65	84.4	
	Yes	30	39.0		Yes	12	15.6	
Loss of grabbing by hands				Loss of grabbing by hands				0.017
	No	57	74.0		No	69	89.6	
	Yes	20	26.0		Yes	8	10.4	
Shoulder pain				Shoulder pain				<0.001
	No	48	62.3		No	67	87.0	
	Yes	29	37.7		Yes	10	13.0	

There was a significant association between shoulder pain recurrence and the type of ACDF. Shoulder pain recurred after one-level ACDF in six (60%) out of 10 patients and only one (10%) experienced shoulder pain after two-level ACDF. No patients who underwent one-level ACDF had this manifestation and five (45.5%) patients experienced it after more than two-level ACDF. Moreover, 11 (55%) of 20 patients complained of left upper limb numbness after two-level ACDF; however, the association was not significant. Table 4 illustrates the recurrence rates of the different manifestations according to the type of ACDF surgery.

**Table 4:** Recurrence of different symptoms after ACDF by type of operation *chi-squared test; **Fisher’s exact test ACDF: anterior cervical discectomy and fusion

	Type of operation						
		One-level ACDF		Two-level ACDF		> Two-level ACDF		P-value
		n=21	%	n=35	%	n=21	%	
Neck pain after ACDF								0.235*
	No	12	21.8	27	49.1	16	29.1	
	Yes	9	40.9	8	36.4	5	22.7	
R upper limb numbness after ACDF								>0.99**
	No	17	27.9	27	44.3	17	27.9	
	Yes	4	25.0	8	50.0	4	25.0	
L upper limb numbness after ACDF								0.572*
	No	16	28.1	24	42.1	17	29.8	
	Yes	5	25.0	11	55.0	4	20.0	
R upper limb pain after ACDF								0.209**
	No	18	27.7	27	41.5	20	30.8	
	Yes	3	25.0	8	66.7	1	8.3	
L upper limb pain after ACDF								0.922**
	No	17	26.2	30	46.2	18	27.7	
	Yes	4	33.3	5	41.7	3	25.0	
Loss of grabbing by hands after ACDF								0.313**
	No	17	24.6	33	47.8	19	27.5	
	Yes	4	50.0	2	25.0	2	25.0	
Shoulder pain after ACDF								0.015**
	No	15	22.4	34	50.7	18	26.9	
	Yes	6	60.0	1	10.0	3	30.0	

## Discussion

Symptoms were significantly reduced after ACDF in this study. Srikhande et al. found similar results, where most symptoms (neck pain, radicular pain, limb weakness, paresthesia, and limb stiffness) improved significantly after surgery [10]. Furthermore, a similar finding was reported in a study of 235 patients, which measured health-related quality-of-life outcomes, such as the visual analog scale (VAS) for neck pain and the neck disability index, and found that both were significantly reduced after ACDF [11]. Our study showed a significant decrease in arm pain after surgery. This was consistent with the findings of Stullet al. [11]. However, 36 four-level ACDF patients displayed a lower, yet insignificant, reduction in the VAS score for arm pain [12]. This could be insignificant because of the minority of patients with predominant radiculopathy compared to our cohort. In the present study, neck pain (28.6%) was the most common recurrent symptom after ACDF. This is consistent with Skrkhande’s 100 participants study, in which the most common recurrent symptoms were neck pain and bladder symptoms (39%) [10].

Regarding the type of ACDF, Vaccaro et al. performed a one-level ACDF on 140 patients and followed them up for 24 months. They found that 72.2%, 67.6%, and 70.4% of the patients developed neck, left arm, and right arm pain, respectively, compared to our one-level ACDF results, which showed 40.9%, 33.3%, and 25% cases of neck, left arm, and right arm pain, respectively [13]. A study conducted by Fountas et al. showed that only 0.2% of patients who received one- to three-level treatment (1,015 patients, 549 men, and 466 women) had their underlying myelopathy deteriorated; most of them (305) had a single-level treatment and only 158 had three-level treatment [14]. This was supported by a study by Flynn, which showed that 0.4% of the patients had deteriorated underlying myelopathy. However, this older study did not mention the cervical level of the surgery [15]. In contrast, Emery et al. and Wang et al. reported the pseudarthrosis rates for non-plated three-level discectomies as 44% and 37%, respectively [16,17]; however, this was only three-level ACDFs. In a study by Tohamy et al. on three-level ACDFs, of 16 patients followed up for 37 months, only seven had postoperative pseudoarthrosis [18]. However, the sample size in this study was small. In our study, 13 of 21 patients showed recurrence. The causes of declining fusion rates with increased operation levels are unclear. It makes sense that the larger the number of surfaces that need to heal, the higher the expected patient-level pseudarthrosis rate. Since these vertebral bodies are vascular, the local blood supply should not be affected by the number of operational levels. Yoo et al. revealed higher contact stress at graft-body interfaces when operational levels increased, supporting the idea that altered biomechanics likely play the most critical role [19].

Regarding the level of cervical procedure, most patients in our study underwent two-level ACDF (35 out of 77 patients); a systemic review reported similar results as our study, with 20 out of 32 patients having the cervical procedure at the C4, C5, and C6 levels [20]. A study by Epstein showed that out of 60 patients, 29 had the procedure done at the C5-C6 and at the C6-C7 level for 20 patients. However, this was a single-level ACDF procedure [21]. In this study, 22 of 24 patients underwent ACDF at the cervical levels C5 or C6, or both [22].

Our study was retrospective, with all the weaknesses and limitations of retrospective studies. This single-center study included all patients; hence, the results cannot be generalized to a larger population. Some files had missing data and were difficult to include, which made our sample smaller. Furthermore, no medications or secondary management was mentioned after the recurrence of symptoms. Additionally, we did not compare ACDF with other procedures.

## Conclusions

ACDF has a high symptom recurrence rate, and the two-level ACDF is the most common type. The most common symptoms were neck and upper limb radicular pain. The C5 level appeared to be the most common cervical level. Studies on symptom recurrence after ACDF are lacking. We recommend doing more studies on secondary management of recurrent symptoms post ACDF. Furthermore, the outcomes of different types of operations should be compared.
